# Automated adherent cell elimination by a high-speed laser mediated by a light-responsive polymer

**DOI:** 10.1038/s42003-018-0222-4

**Published:** 2018-12-07

**Authors:** Yohei Hayashi, Junichi Matsumoto, Shohei Kumagai, Kana Morishita, Long Xiang, Yohei Kobori, Seiji Hori, Masami Suzuki, Toshiyuki Kanamori, Kazuhiro Hotta, Kimio Sumaru

**Affiliations:** 1 iPS Cell Advanced Characterization and Development Team, RIKEN Bioresource Research Center, 3-1-1 Koyadai, Tsukuba, Ibaraki 305-0074 Japan; 2 Kataoka Corporation, 140 Tsukiyama-cho, Kuze, Minami-ku, Kyoto 601-8203 Japan; 3 Meijo University, 1-501 Shiogamaguchi, Tenpaku, Nagoya 468-8502 Japan; 4 Biotechnology Research Institute for Drug Discovery, National Institute of Advanced Industrial Science and Technology (AIST), Tsukuba Central 5th, 1-1-1 Higashi, Tsukuba, Ibaraki 305-8565 Japan; 5 iPS Portal, Inc., 448-5 Kajii-cho, Kamigyo-ku, Kyoto 602-0841 Japan

**Keywords:** Cell culture, Stem-cell biotechnology, Cellular imaging

## Abstract

Conventional cell handling and sorting methods require manual labor, which decreases both cell quality and quantity. To purify adherent cultured cells, cell purification technologies that are high throughput without dissociation and can be utilized in an on-demand manner are expected. Here, we developed a Laser-induced, Light-responsive-polymer-Activated, Cell Killing (LiLACK) system that enables high-speed and on-demand adherent cell sectioning and purification. This system employs a visible laser beam, which does not kill cells directly, but induces local heat production through the *trans*-*cis*-*trans* photo-isomerization of azobenzene moieties. Using this system in each passage for sectioning, human induced pluripotent stem cells (hiPSCs) maintained their pluripotency and self-renewal during long-term culture. Furthermore, combined with deep machine-learning analysis on fluorescent and phase contrast images, a label-free and automatic cell processing system has been developed by eliminating unwanted spontaneously differentiated cells in undifferentiated hiPSC culture conditions.

## Abstract (web summary)

Yohei Hayashi et al. present a method for high-speed adherent cell sectioning and purification, along with a label-free and automatic cell processing system. They show that this method is able to section human induced pluripotent stem cells without losing pluripotency and viability.

## Introduction

The purification of different types of cultured cells is critical in various biomedical fields, including basic research, drug development, and cell therapy. Conventionally, fluorescence-activated cell sorting (FACS), affinity beads (e.g., magnetic-activated cell sorting (MACS)), gradient centrifugation, and elutriation have been used for cell purification^1^. However, these technologies are essentially aimed at floating cells in suspension. For adherent cells, the process of detaching, dissociating, sorting, and reseeding can result in low yield and in altered cell characteristics^2^. For adherent cells, antibiotics or special chemicals have limited use for the selection of genetically modified cells or specialized nutrient-requiring cells, respectively.

To purify adherent cultured cells, in situ cell purification technologies that are high throughput and can be utilized in an on-demand manner are expected. Since light irradiation can be precisely controlled by computers on a microscopic scale and is suitable for sterile processes, methodologies using light have been examined to automate this operation. Among these methods, laser-mediated cell elimination is a promising technology^3,4^. Previous demonstrations, however, revealed only limited success of this method since it requires a high amount of energy to eliminate or move the cells directly, resulting in moderate speed of processing (~1000 cells per second)^3,5–8^. This high auxiliary energy input produces an enormous amount of heat that kills surrounding cells, which destroys the focusing of cell processing. Also, the heat might denature the components in culture media. We previously demonstrated that killing cells through the microprojection of visible light by using photo-acid-generating substrates^9,10^. However, one projection covered only 0.1 cm^2^, and the cell elimination took longer than 1 min in these previous studies.

**Fig. 1:**
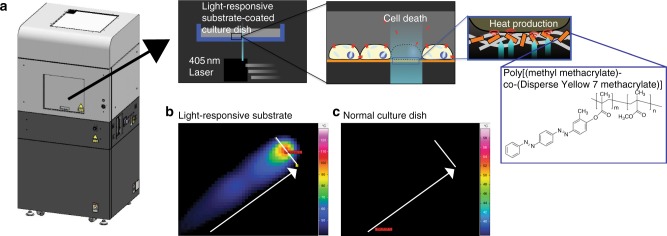
Schemes of the LiLACK system and its focused heat production. **a** Schemes of LiLACK system. **b**, **c** Thermal images of the surfaces of cell culture dishes after laser irradiation. The laser was irradiated at 80 mm per second and 0.3 W with a width of 50 µm towards the arrow direction. The thermal images were acquired in light-responsive polymer-coated dish (**b**) or normal cell culture dish (**c**) from above adjacent without any liquid medium. The bars without arrowheads in the thermally responsive area indicate 50 µm

To overcome these limitations, we have developed a Laser-induced, Light-responsive- polymer-Activated, Cell Killing (LiLACK) system enabling high-speed and on-demand adherent cell sectioning and purification (schemes shown in Fig. 1a). This LiLACK system employs a visible laser beam with a 405 nm wavelength, which does not kill cells directly, but induces local heat production in only the irradiated area of a light-responsive thin layer composed of poly[(methyl methacrylate)-co-(Disperse Yellow 7 methacrylate)]. The energy of the irradiated laser is converted to heat efficiently through the *trans*-*cis*-*trans* photo-isomerization of azobenzene moieties, without photolysis of the polymer^11^. Further, the polymer is free from fluorescence emission and absorbance in most of the visible range, which hinders cell observations. Using this system, human induced pluripotent stem cells (hiPSCs)^12,13^ are sectioned in each passage to maintain their pluripotency and self-renewal in long-term culture. Furthermore, combined with deep machine-learning analysis on fluorescent and phase-contrast images, a label-free and automatic cell processing system has been developed by eliminating unwanted spontaneously differentiated cells in undifferentiated hiPSC culture conditions. This LiLACK system enables to select adherent cells in situ on an acceptable timescale using the precise and very fast scanning of a well-focused visible laser through a light-responsive polymer layer, and automatic label-free cell purification combined with efficient imaging analysis based on deep machine-learning methods.

## Results

### Focused heat production by LiLACK system

First, we examined the effectiveness of local heat production through the *trans*-*cis*-*trans* photo-isomerization of azobenzene moieties. Laser irradiation at 0.3 W and 80 mm per second and with a diameter of 50 µm generated heat at more than 50 °C over focused area of the light-responsive-polymer-coated dishes accurately. In contrast, laser irradiation with the same conditions did not generate detectable heat on the surface of normal cell culture-treated dishes (Fig. 1b, c, and Supplementary Fig. 1). We calculated the distance between the center of the laser spot and end of the tail of comet pattern in the thermal image, which indicated the recovery to a normal physiological temperature. We also confirmed the high-speed temperature changes in the LiLACK system. These results indicated that this scheme enables effective cell killing even at very fast beam scanning without damaging neighboring unirradiated cells. Additionally, we examined the effect of irradiation energy on the temperature of the culture medium. We measured the temperature of the culture media under extreme irradiation conditions in which an entire 35-mm dish was irradiated with the laser at 0.5 W and 80 mm per second at 30-µm intervals (this required approximately 10 min in total). We found that the culture medium increased by only 1.5 °C from room temperature during laser irradiation. These results suggest that heat production by the LiLACK system only affects local areas in the culture medium.

### Growth and viability of hiPSCs on the light-responsive polymer

We examined growth and viability of hiPSC cultured on the light-responsive polymer in both on-feeder^12^ and feeder-free^14,15^ culture conditions. Growth and viability of hiPSCs on the light-responsive polymer were comparable to those on normal cell culture-treated substrates (Supplementary Fig. 2). We also measured the degree of elution of a light-responsive thin layer composed of *trans*-*cis*-*trans* photo-isomerized azobenzene moieties into the culture media by Liquid chromatography–mass spectrometry (LC/MS) methods. We found that the ratio of polymer to decomposition products was below the detection limit (i.e. < 0.1 ppm). These results indicate that the light-responsive polymer does not influence the cultured cell growth and viability.

### Cell killing effectiveness of LiLACK system

**Fig. 2:**
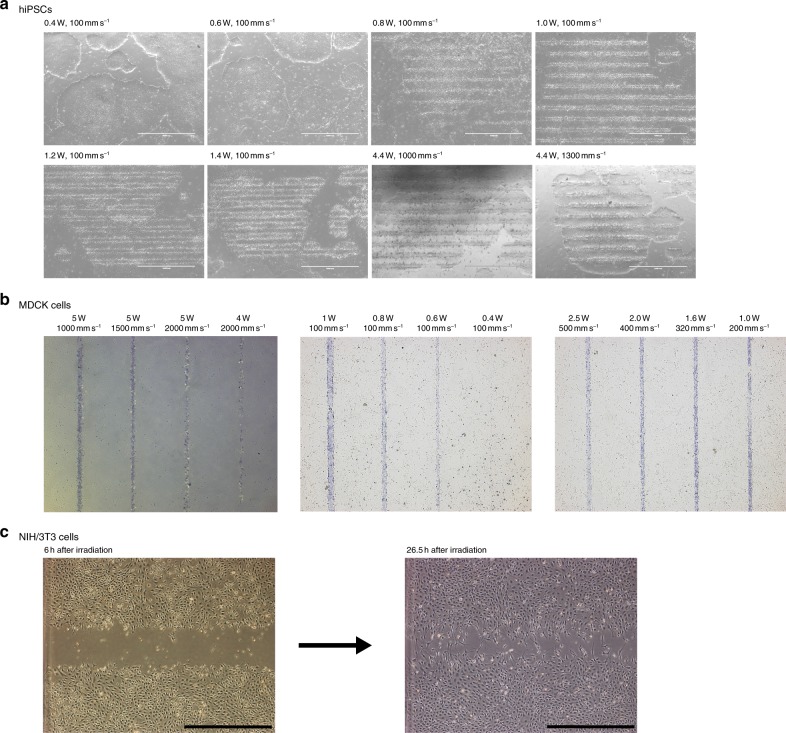
Cell killing of LiLACK system using various cells. **a** Phase-contrast images of hiPSCs after scanning by lasers with different power and speed settings. Scale bars are 1.0 mm. **b** Phase-contrast images of MDCK cells treated with 0.4% trypan blue solution after laser scanning at different power and speed settings. Dead cells were positively stained with trypan blue. The distance between each laser irradiated line was 1 mm. **c** Phase-contrast images of NIH/3T3 cells after laser scanning. The left image was acquired after 6 h, while the right image was acquired after 26.5 h at the same site. Scale bars, 1 mm

We examined the induction of cell death by laser scanning at different power settings and 100 mm per second with a diameter of 50 µm. Laser scanning at a power of 0.8 W or higher readily induced hiPSC death over a diameter of approximately 50 µm (Fig. 2a). Also, using a 4.4 W laser, a scanning speed of 1000 mm per second or higher readily induced hiPSC death. We obtained similar results with the Madin-Darby canine kidney (MDCK) cell line^16^ (Fig. 2b). In the laser scanning at different power setting and 100 mm per second with a diameter of 50 µm, a power of 0.8 W or higher readily induced MDCK cell death over a diameter of approximately 50 µm. Also, the combinations, 2.5 W with 500 mm per second, 2.0 W with 400 mm per second, 1.6 W with 320 mm per second, and 1.0 W with 200 mm per second, which should give the same energy intensity, readily induced similar patterns of MDCK cell death. Using 5.0 W laser, a scanning speed of 1000 mm per second or higher readily induced MDCK death. Assuming that one cell has 10 µm in diameter, we have demonstrated that this LiLACK system enables to process >10,000 cells per second in throughput. Furthermore, over 100,000 cells per second can be processed by the laser irradiation with the higher power laser. These results demonstrate that our LiLACK system enables high-speed and on-demand laser-mediated cell death of various adherent cultured cells. Because we observed that higher laser energy input causes additional cell killing near the scanning line in general, it is critical to input sufficient but the minimum energy to achieve precise cell killing by the LiLACK system.

We also performed laser irradiation on NIH/3T3 cells and examined cellular survival and migration onto the irradiated area. Approximately 1 day after irradiation, the surrounding cells had actively migrated and/or proliferated onto the irradiated area (Fig. 2c). These results suggest that the irradiated area can be reused for cell culture and that this LiLACK system can be used for migration assays in a controlled and automated manner.

### Long-term hiPSC culture using LiLACK system

**Fig. 3:**
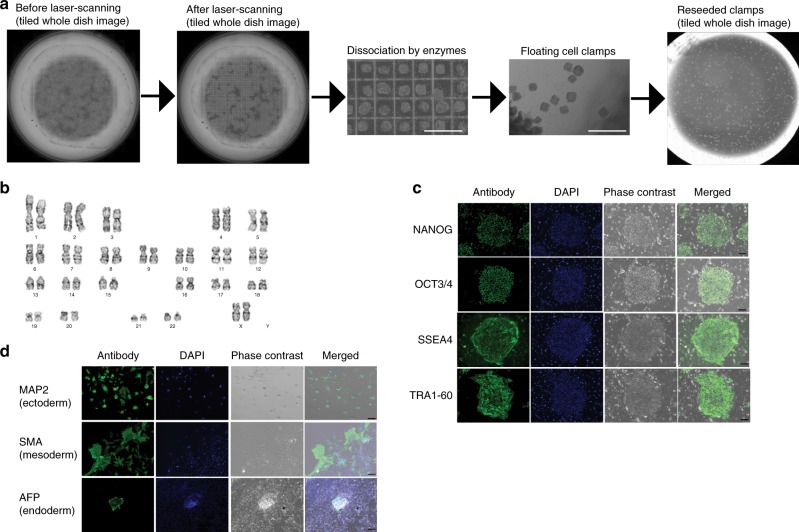
Long-term culture of hiPSCs using LiLACK system. **a** Scheme of the sectioning of a hiPSC monolayer by grid-patterned laser scanning and subcultures. Scale bars, 1 mm. **b** Karyotype example of hiPSCs that underwent ten passages through the system after laser-mediated sectioning. **c** Self-renewal marker expression in hiPSCs that underwent ten passages through the system after laser-mediated sectioning. Scale bars, 100 µm. **d** Expression of three germ layer markers in the embryoid bodies differentiated from hiPSCs that underwent ten passages through the system after laser-mediated sectioning. Scale bars, 100 µm

To examine the feasibility of this LiLACK system in regenerative cell therapies, we examined the effect of the long-term culturing of hiPSCs using LiLACK system. hiPSC colonies cultured on the light-responsive polymer and on feeder cells were cut in a grid pattern by laser scanning and treated with cell dissociation enzyme to generate floating cell clumps (Fig. 3a). The laser beam of 50 µm with 1.0 W intensity was scanned at the velocity 100 mm per second, with 450 µm intervals. Although extra time was needed for the interval between linear scans, the grid scanning took only 86 s to section the cell monolayer in whole area of the 35 mm culture dish surface (9 cm^2^). These hiPSC clumps were transferred to a new light-responsive-polymer-coated culture dish with feeder cells, and the culture continued. We observed homologous iPSC colonies in their size, which were generated from processed clumps, as previously demonstrated^7,10^. After ten passages through the system consisting of the above steps, the hiPSCs were characterized. The karyotype of these hiPSCs was maintained in all the cells (i.e., 50 cells per 50 cells) (Fig. 3b). These hiPSCs expressed self-renewal markers of pluripotent stem cells, such as NANOG, OCT3/4, SSEA4, and TRA1-60, which were determined immunocytochemically (Fig. 3c). When differentiated into three germ layers using embryoid body formation, these cells expressed, microtubule-associated protein 2 (MAP2) as an ectoderm marker, smooth muscle actin (SMA) as a mesoderm marker, and α-fetoprotein (AFP) as an endoderm marker, detected by immunocytochemistry (Fig. 3d). Additionally, these hiPSCs were free of harmful viruses and mycoplasma detected by quantitative PCR-based tests (Supplementary Table 1). These results indicate that our LiLACK system using laser-mediated cell processing maintained the self-renewal, pluripotency, and biological safety of hiPSCs during long-term culture.

### Deep-learning analysis to identify spontaneously differentiated hiPSCs

**Fig. 4:**
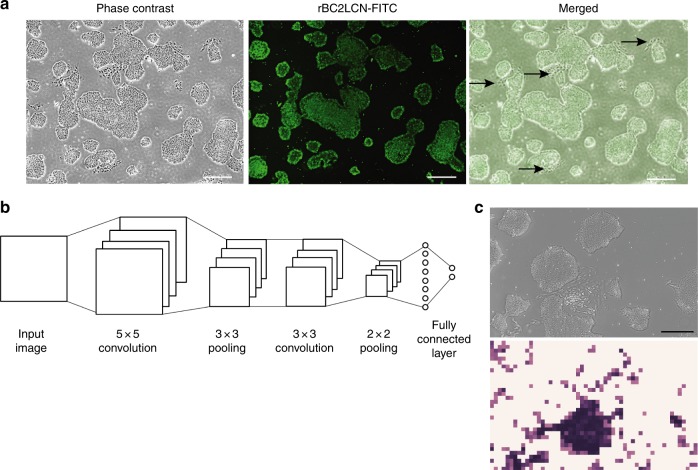
Deep machine learning to identify spontaneously differentiated hiPSCs. **a** Example of the “teacher” image sets used in the machine learning. Live-cell phase-contrast and fluorescence images from the undifferentiated marker probe (rBCN2CL-FITC) were combined. Arrows indicate the spontaneously differentiated cells that were rBCN2CL-negative. Scale bars, 200 µm. **b** Scheme of the CNN structure. **c** Example of an input image (upper) and output image (bottom) generated by the algorithm generated from the CNN results. In the output image, dark areas indicate differentiated cells automatically classified from the input image. Scale bar, 200 µm

Because this LiLACK system enables cell processing based on phase-contrast and fluorescent cell morphologies, we developed a label-free cell elimination system based on deep machine-learning imaging analysis. Emerging spontaneous differentiated cells in undifferentiated human pluripotent stem cell culture conditions is important problems in obtaining reproducible and reliable results in basic scientific studies and therapeutic cell production^17–19^. We tried to solve this problem by automatically eliminating these differentiated cells using this LiLACK system combined with deep-learning imaging analysis. First, we collected phase-contrast and fluorescence images using a fluorescent probe, rBC2LCN-FITC, to mark undifferentiated hiPSCs^20,21^ (example in Fig. 4a). Using the LiLACK device, which carries the function to collect tiled images of a whole dish, 12,556 differentiated images and 18,834 undifferentiated images were collected. These images were used to train a convolutional neural network (CNN). Figure 4b shows the structure of the CNN. The size of an input image was set to 70 × 70 pixels. Since the size of an original image is 1920 × 1200 pixels, local regions of 70 × 70 pixels can be cropped without overlap, and the regions can be fed into the CNN. Our CNN consisted of two convolutional layers, two pooling layers, and a fully connected layer. Thirty-two filters with 5 × 5 and 3 × 3 kernel sizes were used in the first and second convolutional layers, respectively. After the convolutional layers, we used a maximum pooling with 3 × 3 and 2 × 2 kernel sizes. The softmax cross entropy loss was used to train the CNN. Undifferentiated images were gathered in two ways. First, 70 × 70 local regions were cropped randomly from differentiated and undifferentiated regions, and the CNN was trained by those images. The trained CNN was applied to images, and misclassified undifferentiated regions were gathered. The gathered undifferentiated cell images were added to the training images, and the CNN was trained again and used as the classifier. Figure 4c shows the original image and the detection result from the CNN. The probability of obtaining a differentiated class from the CNN is assigned to the pixel in the resulting image. The black pixel in the resulting image shows the differentiated region. The intensity of the black color reflects the probability: darker colors indicate higher probability. These results indicated that deep machine-learning imaging methods were able to distinguish spontaneously differentiated cells in undifferentiated hiPSC culture conditions.

### Automatic in situ purification of undifferentiated hiPSCs using LiLACK system

**Fig. 5:**
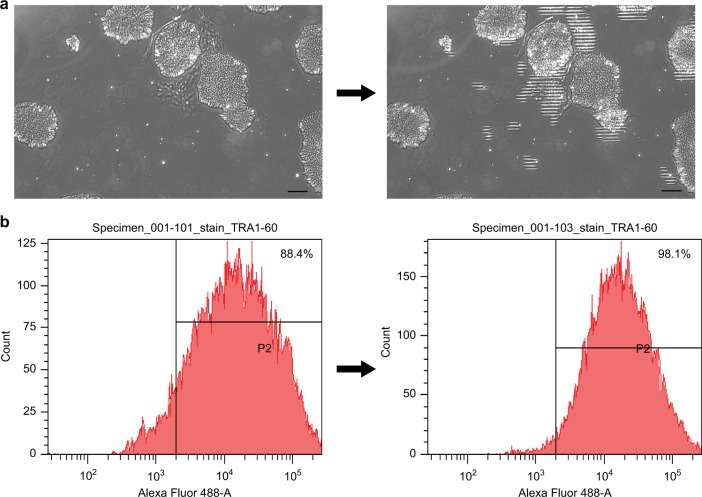
In situ automatic purification of undifferentiated hiPSCs using LiLACK system. **a** Example of the automatic laser-mediated elimination of spontaneously differentiated cells in normal hiPSC culture conditions. Scale bars, 100 µm. **b** Flow cytometric analysis of TRA1-60, a cell surface pluripotency marker, expression in hiPSCs with or without laser-mediated cell elimination of spontaneously differentiated cells

Last, we applied this trained classification algorithm to our laser-mediated cell elimination with only phase-contrast images (Fig. 5a and Supplementary Movie 1). The laser beam of 50 µm with 0.8 W intensity was scanned at the velocity 100 mm per second, with 25 µm intervals; these conditions were determined be effective for killing cells at the lowest energy. The laser irradiation was activated only when the beam was in the target areas. Purification in whole area of the culture surface took 667 s, which could be shorten according to the distribution of the target cells. hiPSCs, which were treated with automated cell elimination or not, were collected and analyzed for TRA1-60-positive cells, which indicated undifferentiated hiPSCs^22,23^, by flow cytometry analysis. TRA1-60-positive cell ratio increased to 97% or more after laser irradiation to eliminate the “differentiated” cells classified by this algorithm (Fig. 5b and Supplementary Fig. 3). These results indicate that in situ label-free cell purification was automatically achieved using our LiLACK system combined with imaging analysis based on deep learning.

## Discussion

We have developed a novel system for selecting adherent cells in situ on an acceptable timescale using the precise and very fast scanning of a well-focused visible laser through a light-responsive polymer layer at 100 mm per second or more ( >10,000 cells per second). Combined with efficient imaging acquisition and analysis based on machine learning, we achieved automatic label-free cell purification and sectioning. Compared with conventional methods, such as FACS, affinity beads, gradient centrifugation, elutriation, and direct laser-induced cell killing (summarized in Table 1), our LiLACK system may have advantages in maintaining the cell numbers and characteristics since our methods do not directly affect the unirradiated cell activities. Thus, we believe that our methods can be widely used in various biomedical fields, including basic research, drug development, and cell therapy.

**Table 1:** Summary of the comparisons between conventional cell purification systems and our new LiLACK system

Selection type	FACS	Affinity beads	Gradient centrifugation	Antibiotics-based cell separation	Direct laser cell killing system	(Indirect) LiLACK system
Cell preparation	Dissociation	Dissociation	Dissociation	Genetic modification with antibiotics-resistant genes or cell type specific chemicals	No need (or labeled by fluorescent probes)	No need (or labeled by fluorescent probes)
Culture condition needed	None	None	None	Specific antibiotics or chemicals	Specific dye and only glass dishes	Specific light-responsive-substrate-coated dishes
Throughput	~1000 cells per second	~10 million cells per second	~30 min per centrifuge	Several days or weeks	~1000 cells per second	>10,000 cells per second
Device	FACS (BD, Beckman Coulter and others)	Magnetic Sorting Device (Miltenyl Biotec and others)	Centrifuge and gradient reagents (many manufacturers)	None	LEAP (Intrexon) and others	Specific Laser-scanning device (Kataoka Corp)

Regarding the mechanistic basis of the LiLACK system, we employed a light-responsive polymer that has been used for non-linear optics. We expected to observe reduced decomposition caused by side reactions and fluorescence. The azobenzene structure of the polymer was previously demonstrated to exhibit fast continuous *trans*–*cis*–*trans* cycling following excitation at one wavelength^24^. Photon absorption does not always lead to structural changes, which can be partially explained by the simple absorption of light, followed by excitation and then relaxation to the ground state while converting light energy into heat. Using this polymer, we suppressed decomposition occurring through side reactions and fluorescence.

This system also enables high-speed cell killing with local temperature changes. At a typical scanning velocity of 100 mm per second, the laser beam of ⌀ 50 µm passes a cell (~10 µm) in approximately 0.5 ms. The order of the thermal diffusion coefficient of the system is 0.1mm^2^ per second, suggesting that the heat diffuses a few tens of micrometers in 10 ms. Based on these estimations, the local temperature increases in 1 ms and then decreases over 10 ms. Indeed, as shown in Fig. 1b, the snapshot of thermal image at a 200-Hz frame rate (the interval of the image was 5 ms) of laser irradiation at 80 mm per second with a width of 50 µm indicated an acute temperature increase at the laser spot and rapid temperature decrease in the light path. We calculated the distance between the center of the laser spot and end of the tail of “comet” pattern of the thermal image, which indicated the recovery to a normal physiological temperature. This result confirmed the high-speed temperature changes in the LiLACK system. This fast and local temperature cycling also results in minimal cell killing near the scanning area.

The LiLACK system should be particularly suitable for use in cell therapies in future. Because our LiLACK system does not require direct manual handling to process cells and can be easily operated in an isolated space or even in an incubator for cultured cells in principle, it can reduce risk of contamination or exposure to hazardous substances compared with the risks associated with conventional cell sorting using FACS, MACS, or other filtering methods. Additionally, compared with previous reports using specific dyes supplemented in the cell culture medium for the efficient energy absorption of laser irradiation^3–7^, our methods do not produce free chemicals in the culture medium. Although the light-responsive polymers used in this method should be thoroughly scrutinized for biological safety, such as cytotoxicity and carcinogenicity, the LiLACK system has great potential for use in cell therapies, with the capability for real-time monitoring and in situ automated cell processing combined with imaging analysis based on machine learning.

To achieve label-free cell purification of undifferentiated hiPSCs based on the colony morphology observed in phase-contrast images, we utilized a deep-learning-based classification of undifferentiated and differentiated hiPSCs. Previous studies demonstrated the utility of morphological analysis to monitor the quality of hiPSCs^25,26^. In recent years, the effectiveness of deep machine learning for image recognition has been demonstrated. CNNs have worked well in various applications, such as object categorization^27^, object tracking^28^, action recognition^29^ and particle detection in intracellular images^30^. Therefore, we used a CNN to classify differentiated cells and undifferentiated cells in an unbiased manner. We successfully demonstrated that undifferentiated hiPSCs were enriched by the automated cell killing system based on the CNN-predicted program to identify spontaneously differentiated cells. This demonstration indicates that the hiPSC quality could be continuously refined from non-labeled images combined with our laser-processing technology. Since our technology can be applied to any adherent cells and does not usually interfere with the fluorescence from labeled proteins used for various biological purposes, we believe that our technology is compatible with the purification of various fluorescent reporter cells, as well as non-labeled cells. Since the classification of cell types based on deep-learning methods is advancing rapidly, the importance and functionality of our technology will be further enhanced in the near future.

In summary, we demonstrated laser cutting of hiPSCs into pieces for passaging and removal of spontaneously differentiated cells in one procedure for hiPSC maintenance. Because both applications can be combined, these techniques can be used to develop a fully automated culture system for human PSCs to ensure the quality and homogeneity of the cells. This LiLACK system can be applied to improve the purity of target differentiated cells from stem cells by removing unwanted differentiated cells or directing the cells to another lineage.

## Methods

### Fabrication of photoresponsive culture substrates

The photoresponsive polymer used in this study was poly[(methyl methacrylate)-co-(Disperse Yellow 7 methacrylate)] (#579149, Sigma-Aldrich), which was composed of ~25 mol% azobenzene moieties. The polymer was dissolved in a mixture of 2,2,2-trifluoroethanol and 1,1,1,3,3,3-hexafluoro-2-propanol (10 wt%) to form a 1.0 wt% solution. Next, 20 µL of the solution was spin-coated on a polystyrene cell culture dish with a 35 mm diameter (#3000-035, AGC Techno Glass, Japan) at 2000 rpm under an N_2_ atmosphere, and then the substrates were annealed for 2 h at 80 °C. The absorbance of the substrate was typically 0.25 at 405 nm and less than 0.05 at wavelengths greater than 500 nm.

### Laser irradiation

Laser irradiation was conducted at a specific energy [W] and speed [mm per second] with a diameter of 50 µm (except where specified). We defined [W] using laser power “P” and the speed of linear laser irradiation “v”, respectively. When P[W] and v[mm per second] are defined, the irradiated energy to 1 mm length is P per v [J per mm]. For example, when the laser is irradiated with 1 W at 100 mm per second, the irradiated energy is 0.01 J per mm. We assumed that the irradiated laser was 50 µm wide, which is the same value as the input. Thus, the energy flux was 0.2 J per mm^2^. Although the cross-sectioned laser is round-shaped, the thermal diffusion of this laser system is approximately 0.1 mm^2^ per second. Thus, this assumption is practically acceptable.

### Laser scanning device

The laser beam was irradiated from a semiconductor laser source device through an optical fiber waveguide to the exit lens attached to an optical driving system. Laser scanning was performed by motor-driven regulation of the exit lens at a coordinate axis of the horizontal direction with high-speed and accuracy. The laser was focused at the surface of the cell-substrate interaction by the optical driving system to achieve efficient photothermal conversion by the light-responsive-polymer. The composition of the phase-contrast microscopy system was generally set with a ring slit and objective lens carrying a phase plate. Microscopy images were acquired with a CMOS (complementary metal-oxide-semiconductor) camera. The ON/OFF of the laser irradiation was regulated by the input electricity of the semiconductor laser and was adjusted to the laser scanning point. The position of switching the electricity was determined in concordance with the coordinates of the phase-contrast microscopy and laser device coordinates.

### Thermal imaging

Thermal images were taken by an infrared thermography camera, InfReC H9000 (Nippon Avionics, Japan) with a 5 µm microscopy lens with the following measurement parameters: wavelength, 2–5.7 µm; temperature, –10 to 1200 °C; temperature resolution, 0.025 °C at 30 °C; frame rate, 200 Hz; spatial resolution, 5 µm; and temperature accuracy, 2% at an environmental temperature of 10–40 °C. The laser irradiated a cell culture dish coated with or without the light-responsive substrate at an environmental temperature of 29.5 °C without any liquid medium. The laser conditions were 0.3 W to 0.7 W at 80 mm per second.

### Liquid chromatography–mass spectrometry

The degree of elution of a light-responsive thin layer composed of *trans*-*cis*-*trans* photo-isomerized azobenzene moieties into the culture media was measured by LC/MS methods (Acquity TQD, Waters). The ratio of polymer to decomposition products was below the detection limit (i.e.,< 0.1 ppm). This LC/MS assay were performed at Kyoto Municipal Institute of Industrial Technology and Culture.

### Cell processing by laser scanning

To achieve high-speed, automatic cell processing using 405 nm laser scanning, we developed an all-in-one apparatus that can take phase-contrast and fluorescence images and apply tiled, whole-dish laser scanning in an on-demand manner (Kataoka Corp, Japan; Fig. 1a). First, culture dishes are selected from a cell culture incubator, and photographs of the whole dish are taken and analyzed. Then, the type (e.g., lattice pattern, rectangle, circle, and line) and conditions (e.g., speed and power) of laser scanning are selected. Once the conditions are determined, the on-demand laser irradiation is initiated. The irradiated culture dishes are then re-incubated and subjected to assays. A demonstration of the procedure is shown in Supplementary Movie 1.

### MDCK cell culture

An MDCK cell line was purchased from the RIKEN Bioresource Research Center. The cells were maintained in Eagle’s minimum essential medium (Sigma-Aldrich) supplemented with 10% fetal bovine serum (Gibco BRL). For passage through the system, the culture medium was removed and discarded. The cell layer was rinsed twice with a 0.25% (w per v) trypsin-0.53 mM EDTA solution to remove all traces of serum that contained trypsin inhibitor. The trypsin-EDTA solution was added to the dish, and the cells were observed under an inverted microscope until the cell layer dispersed (usually within 5 to 15 min). Complete growth medium was added, and the cells were aspirated by gently pipetting. Appropriate aliquots of the cell suspension were added to new light-responsive-polymer-coated dishes. The cells were incubated at 37 °C and 5% CO_2_ in an incubator. The medium was replaced every 2 to 3 days. Laser irradiation experiments were performed on fully confluent conditions.

### hiPSC culture

hiPSCs (201B7 or 1231A3 lines)^12^ were obtained from CiRA (Center of iPS Cell Research and Application) at Kyoto University through the RIKEN Bioresource Research Center (Tsukuba, Japan). All media and reagents were purchased from commercial sources. For on-feeder culture conditions, the cells were maintained in Primate ES cell medium (Reprocell, Tokyo, Japan) or Stemsure on-feeder hPSC medium (Wako, Osaka, Japan) supplemented with 4 ng per ml bFGF and penicillin/streptomycin (Nacalai Tesque) on SNL feeder cells, which were from a mitomycin C (Sigma-Aldrich)-treated SNL 76/7 cell line (Cell Biolabs) described previously^31–35^. For subculturing, the cells were detached from the culture dish using CTK solution (Reprocell)^36^. The SNL cells were cultured in fibroblast medium (Dulbecco’s Modified Eagle Medium (DMEM) supplemented with 10% fetal calf serum, 1% penicillin, and 1% streptomycin) on gelatin-coated dishes. These cell clumps were transferred to a new light-responsive-polymer-coated dishes on SNL feeder cells at 1:3 to 1:10. For feeder-free culture conditions, the cells were maintained in StemFit AK02N medium (Ajinomoto, Tokyo, Japan) supplemented with all the abovementioned supplements on 0.5 µg per cm^2^ iMatrix511 (Nippi, Japan)-coated dishes^14,15,37^. A Rho-associated protein kinase (ROCK) inhibitor (Y-27632, Wako, Osaka, Japan) (10 µM) was added to the medium used in the passage process^38^. For subculturing, the hiPSCs adhered to a culture dish were washed with phosphate buffered saline (PBS) and then treated with TrypLE Select (Thermo Fisher Scientific) in Dulbecco’s PBS at 37 °C for approximately 5 min. After aspirating the solution, the cells were resuspended in fresh medium. Then, the cells were collected by cell scraping and pipetting. After counting the cell numbers, the cells were normally seeded at 2.5 × 10^3^ cells per cm^2^.

### NIH/3T3 cell culture

NIH/3T3 cells (mouse embryonic fibroblasts) were purchased from RIKEN Bioresource Research Center (Tsukuba, Ibaraki, Japan) and cultured in DMEM (#041-29775, Wako Pure Chemical Industries) containing 10% fetal bovine serum (FBS, #30-2020, ATCC). Laser irradiation experiments were performed 1 day after seeding.

### Immunocytochemistry

For the immunocytochemical determination of pluripotency and differentiation markers, cells were fixed with PBS containing 4% (vol per vol) paraformaldehyde for 10 min at room temperature. The cells were permeabilized with PBS containing 0.1% Triton X-100 for 10 min at room temperature, then washed with PBS and treated with 1% BSA for blocking. For hiPSCs or reprogramming HDFs, the primary antibodies used were SSEA4 (0.5 µg per mL; eBiosciences), TRA-1-60 (0.5 µg per mL; eBiosciences), NANOG (2 µg per mL, AF1997; R&D Systems), OCT3/4 (1:200, sc-5279; Santa Cruz Biotechnology), MAP2 (1:1000, AB5622; Millipore), α-SMA (1:1000, A2547; Sigma), and AFP (2 µg per mL, MAB1368; R&D Systems). The secondary antibodies used were Alexa Fluor 488- or 555-conjugated goat anti-mouse IgG (1:200; Invitrogen), Alexa 488- or 555-conjugated goat anti-rabbit IgG (1:200; Invitrogen), and Alexa 488- or 555-conjugated donkey anti-goat IgG (1:200; Invitrogen). Nuclei were stained with the 4’,6-diamidino-2-phenylindole (DAPI) contained in the VectaShield set (Vector Laboratories). The samples were analyzed in randomly selected images with BZ-X710 (Keyence, Osaka, Japan).

### hiPSC differentiation

Embryoid bodies were generated by CTK medium treatment of day-7 hiPSC cultures to remove colonies from the culture dishes. Colonies were grown in differentiation medium in a suspension culture using EZ-BindShut II dishes (Iwaki, Japan). hiPSCs were induced to spontaneously differentiate in a medium composed of StemSure DMEM (Wako) supplemented with 20% StemSure serum replacement (Wako), 2 mM l-alanyl-l-glutamine (Wako), 1% MEM non-essential amino acids (Wako), and 0.1 mM 2-mercaptoethanol (Thermo Fisher Scientific). EBs were grown in suspension culture for 8 days, then plated onto gelatin-coated plates and allowed to differentiate for an additional 8 days in DMEM + 10% FCS.

### Karyotyping

Chromosomal G-band analyses were performed at Nihon Gene Research Laboratories Inc.

### Image acquisition and CNN analysis

Phase-contrast and fluorescence images labeled with rBC2LCN-FITC (Wako, Osaka, Japan) were taken by the automatic laser-processing device (Kataoka Corp, Kyoto, Japan). Twelve-thousand five-hundred fifty six differentiated images and 18,834 undifferentiated images were used to train a CNN. The size of an input image was set to 70 × 70 pixels. Since the size of an original image is 1920 × 1200 pixels, local regions of 70 × 70 pixels can be cropped without overlap, and the regions can be fed into the CNN. Our CNN consisted of two convolutional layers, two pooling layers, and a fully connected layer. Thirty-two filters with 5 × 5 and 3 × 3 kernel sizes were used in the first and second convolutional layers, respectively. After the convolutional layers, we used a maximum pooling with 3 × 3 and 2 × 2 kernel sizes. The softmax cross entropy loss was used to train the CNN. Undifferentiated images were gathered in two ways. First, 70 × 70 local regions were cropped randomly from differentiated and undifferentiated regions, and we trained the CNN using those images. The trained CNN was applied to images, and misclassified undifferentiated regions were gathered. The gathered undifferentiated cell images were added to the training images, and the CNN was trained again and used as the classifier. The probability of obtaining a differentiated class from the CNN is assigned to the pixel in the resulting image.

### Automatic cell purification using LiLACK system

hiPSCs (201B7 cell line) cultured in feeder-free conditions on light-responsive substrate-coated 35 mm dishes (Iwaki) for 7 days were used for laser irradiation. First, whole-dish tiled images were taken by the LiLACK device (Kataoka Corporation) and were analyzed by the algorithm made by the CNN analysis described above. Then, the laser was irradiated to the area automatically determined as differentiated cells by the algorithm. All the cells in the treated dish were used for flow cytometry analysis.

### Flow cytometry

Harvested cells were fixed with PBS containing 4% (vol per vol) paraformaldehyde for 10 min at room temperature. Then, the cell samples were washed with PBS containing 0.5 mM EDTA and 1% (vol per vol) FBS and stained with mouse monoclonal anti-TRA1-60 antibodies, clone TRA1-60, Alexa Fluor 488 conjugate (Merck Millipore; MAB4360A4), for 1 h at 4 °C. Cells were then washed three times with PBS containing 0.5 mM EDTA and 1% FBS. After washing, cells were filtered through a 70 mm cell strainer (BD). The stained cells were analyzed with FACSAria II (BD) and FACSDiva software (BD).

### Determining the cell numbers and viability

The cell numbers and viability were examined using an automated cell counter (NucleoCounter NC-200, Chemometec). Briefly, total and dead cell numbers were counted by staining with acridine orange and DAPI, respectively.

### Viruses and mycoplasma infection tests

Viruses and mycoplasma infection tests, using multiplex quantitative PCR methods, were performed in PharmaBio Corporation.

### Code availability

The custom code can be available in the Supplementary Software 1. The license of use with restrictions will be provided by Meijo University and Kataoka corporation.

## Electronic supplementary material

Supplementary Information

Description of Additional Supplementary Files

Supplementary Movie 1

Supplementary Software 1

## Data Availability

All data generated or analyzed during this study are included in this published article (and its supplementary information files).
