# Supplementary material for: Automated adherent cell elimination by a high-speed laser mediated by a light-responsive polymer
Source: Commun Biol. 2018 Dec 7;1:218. doi: 10.1038/s42003-018-0222-4 (PMC6286311; doi:10.1038/s42003-018-0222-4)
Supplement: Supplementary file 1 — Supplementary Information [file 42003_2018_222_MOESM1_ESM.pdf]

Supplementary Information

Supplementary Figure 1

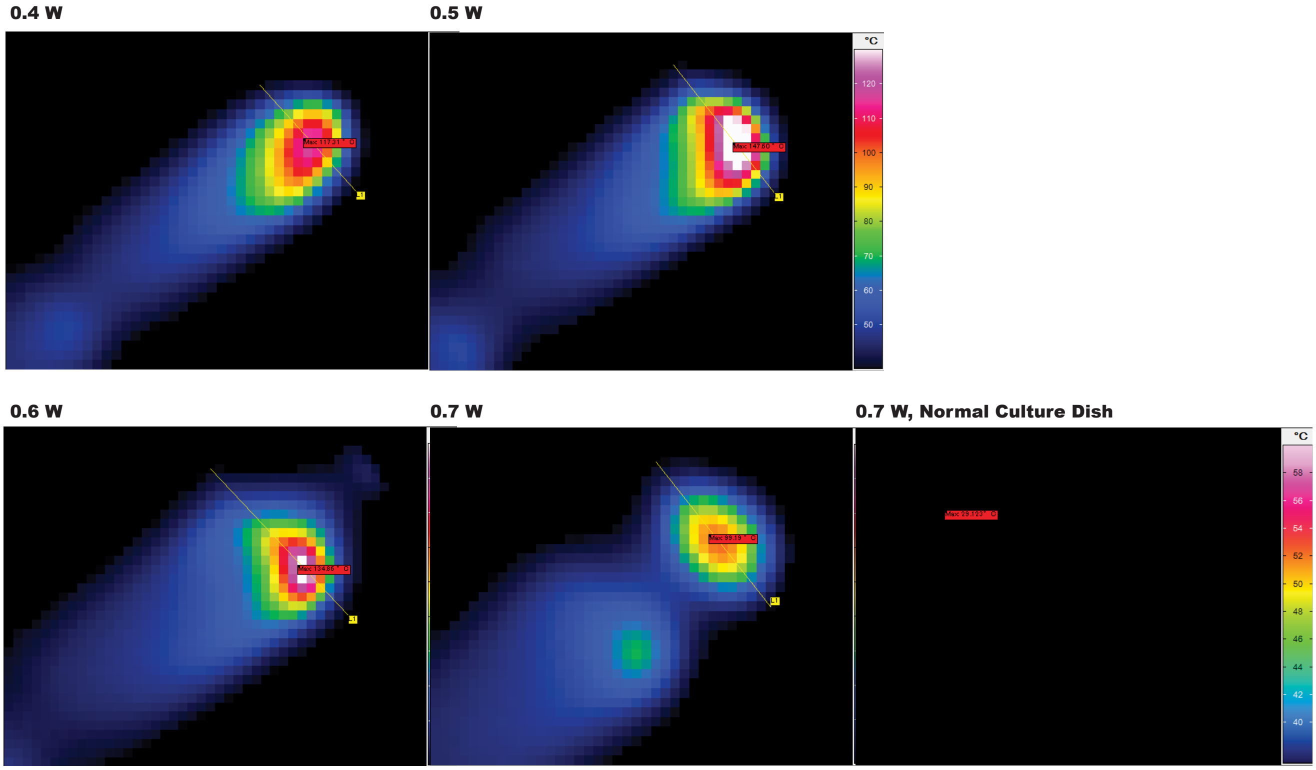

Supplementary Figure 1. Thermal images of the surfaces of cell culture dishes coated with the light-responsive-polymer or normal culture dishes right after laser irradiation. Laser irradiation occurred at 80 mm s^{-1} with a power range of 0.4 W to 0.7 W.

Supplementary Figure 2

a

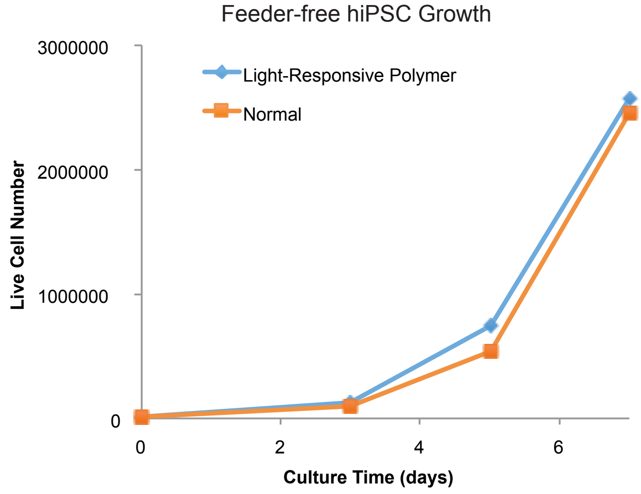

Cell Viability	Day 3	Day 5	Day 7
Light-responsive	78.8%	83.0%	92.3%
Normal	63.0%	70.7%	60.9%

b

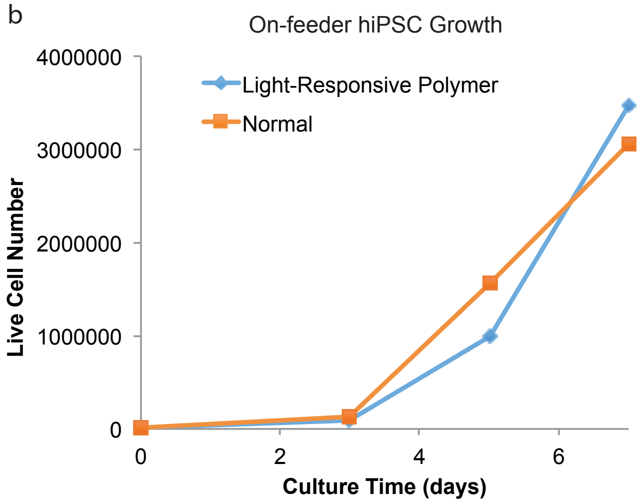

Cell Viability	Day 3	Day 5	Day 7
Light-responsive	90.1%	85.0%	93.8%
Normal	95.1%	95.4%	93.4%

Supplementary Figure 2. Cell growth of hiPSCs on the light-responsive polymer in feeder (a) and feeder-free (b) culture conditions. Cell numbers and viability were calculated using an automated cell counter by staining with acridine orange and DAPI.

Supplementary Figure 3

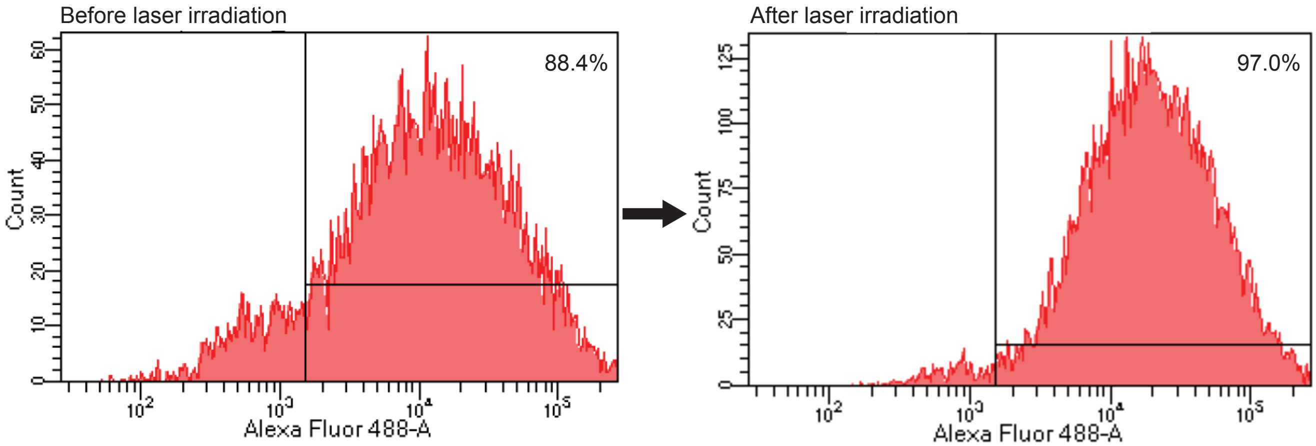

Supplementary Figure 3. Flow cytometric analysis of TRA1-60, a cell surface pluripotency marker, expression in hiPSCs with or without laser-mediated cell elimination of spontaneously differentiated cells (other experimental settings were the same as those in Figure 5b).

Supplementary Table 1

Type	Result
HIV1 DNA	Negative
HIV1 RNA	Negative
HIV2 DNA	Negative
HIV2 RNA	Negative
HTLV1 DNA	Negative
HTLV1 RNA	Negative
HTLV2 DNA	Negative
HTLV2 RNA	Negative
HCV RNA	Negative
HBV DNA	Negative
EBV DNA	Negative
CMV DNA	Negative
Parvo19 DNA	Negative
Mycoplasma	Negative

Supplementary Table 1. Results of viral and mycoplasma infection tests in hiPSCs that underwent 10 passages through LILACK system with laser-mediated sectioning.
