# Supplementary material for: Automated adherent cell elimination by a high-speed laser mediated by a light-responsive polymer
Source: Commun Biol. 2018 Dec 7;1:218. doi: 10.1038/s42003-018-0222-4 (PMC6286311; doi:10.1038/s42003-018-0222-4)
Supplement: Supplementary file 2 — Description of Additional Supplementary Files [file 42003_2018_222_MOESM2_ESM.docx]

**Description of Additional Supplementary Files**

**File Name**: Supplementary Movie 1

**Description**: The demonstration of LILACK system for automatic hiPSC recognition and purification.

**File Name**: Supplementary Software 1

**Description**: A Python code for training Convolutional Neural Network.
